# Karnofsky Performance Scale validity and reliability of Turkish palliative cancer patients

**DOI:** 10.3906/sag-1810-44

**Published:** 2019-06-18

**Authors:** Nagihan YILDIZ ÇELTEK, Mustafa SÜREN, Osman DEMİR, İsmail OKAN

**Affiliations:** 1 Department of Family Medicine, Faculty of Medicine, Tokat Gaziosmanpaşa University, Tokat Turkey; 2 Department of Anesthesiology and Reanimation, Faculty of Medicine, Tokat Gaziosmanpaşa University, Tokat Turkey; 3 Department of Biostatistics, Faculty of Medicine, Tokat Gaziosmanpaşa University, Tokat Turkey; 4 Department of General Surgery, Faculty of Medicine, Tokat Gaziosmanpaşa University, Tokat Turkey

**Keywords:** Palliative care, Karnofsky Performance Scale, quality of life

## Abstract

**Background/aim:**

Validated measures in palliative cancer patients are very important in the evaluation and management of the disease. The Karnofsky Performance Scale (KPS) has been used for many years to assess the performance status of cancer patients. The aim of this study is to determine the validity and reliability of the KPS in cancer patients receiving palliative care in Turkey.

**Materials and methods:**

Eighty patients with a cancer diagnosis who were admitted to Gaziosmanpaşa University Medical Faculty Hospital Palliative Care Unit between 01.03.2016 and 01.03.2017 were included in the study. KPS, measurements from the Katz Activities of Daily Living (ADL) scale, and Basic Activities of Daily Living (BADL) scale were recorded. The alpha coefficient (Cronbach) was calculated by using SPSS version 20.0. The P-value was accepted as P  < 0.05 in the analysis of the data.

**Results:**

There was a positive and strong correlation between Katz ADL scale total score and KPS score (r = 0.895; P < 0.001). In addition, there was a strong negative correlation between the total score of BADL scale and KPS score (r = –0.894; P < 0.001). As for the reliability of the scale scores, Cronbach’s alpha coefficient found to be 0.720.

**Conclusion:**

KPS is a reliable scale for Turkish cancer patients in palliative care settings.

## 1. Introduction

Palliative care is an integral part of cancer care. It involves a prompt and holistic evaluation of patients, including their physical, social, and spiritual needs. Although the need for palliative care cancer patients can be identified in the early stages of the disease, it becomes more prominent as the patient reaches the end of life [1]. Palliative care services can be provided in outpatient clinics, hospitals, specialized care centers, hospice centers, or at patients’ homes by home care units [2]. To provide an effective palliative care service, the patient’s symptoms and physical performance status should be assessed accurately. Performance status assessment is crucial for the evaluation of general well-being of cancer patients since it provides an insight into the general physical condition of the patient which is the basis for the advanced treatment decision. Performance assessment is also used to measure patients’ quality of life [3,4]. There are various scales used for the evaluation of palliative care patients. The Karnofsky Performance Scale (KPS), the Edmonton Symptom Assessment Scale, the Katz Index of Independence in Activities of Daily Living (Katz ADL), the Palliative Performance Scale, the Palliative Prognostic Score, and the Palliative Prognostic Index are commonly used scales [5].

The KPS was defined by Dr. Joseph H. Bruchenal and Dr. David A. Karnofsky in 1949. The KPS is widely used throughout the world for performance assessment of cancer patients [4,6]. The functional status of a patient is assessed on an 11-point scale ranging from full well-being (100%) to death (0%), decreasing ten points at each level. According to the assessment results, patients are divided into three groups; Group A (100%–80%) can independently perform daily activities, Group B (70%–50%) can perform daily activities with help, Group C (<40%) requires continuous assistance and approaches death progressively [2,7].

Although palliative care-related studies have been published for a while from Turkey, thoroughly institutionalized palliative care services started to be established in 2013. Since palliative care is in the early stage of development in our country, studies designed to reveal the patient profile and characteristics are needed. The validation of well-known scales and measures used for palliative patients throughout the world in our settings with our patients would enable us to evaluate them more accurately and compare our services with the rest of the world. 

Here, we aimed to validate the KPS in palliative care settings with Turkish patients.

## 2. Materials and methods

The study’s sample was comprised of patients diagnosed with cancer and receiving follow-up treatment in the Palliative Care Unit at Gaziosmanpaşa University Medical Faculty Hospital. Our unit was founded in October 2015. A total of 80 patients referred to the Palliative Care Outpatient Clinic between 01.03.2016 and 01.03.2017 were included. With the help of the G*power 3.1.2 program, the sample size was determined as 80 with a power of 80%, type I error of 5%, and an effect size of 0.282. Between the dates mentioned above, 820 patients applied to our unit for examination and treatment. The standard patient group included in the study was the patients between 18 and 90 years of age. Patients who did not want to participate in the study and those with communication problems were excluded. Patients included in the study were informed and their consent was taken. Ethical committee approval for the study was also obtained (Tokat Gaziosmanpaşa University Clinical Research Ethical Committee/19.01.2016/16-KAEK-012). The KPS was translated from English into Turkish by the academics working in the Palliative Care Working Group (1 member of the Department of Anesthesiology, 1 member of the Department of General Surgery, and 2 members of the Department of Public Health) and made applicable. On this scale, a patient’s general condition is scored from 0 to 100; 100 means that the performance status is very good, i.e. they are healthy, and 0 indicates the death of the patient. Each 10-point decrease on the scale means that the patient’s condition is getting worse.

The Katz ADL consists of 6 questions, including information about bath-taking, self-dressing, restroom, mobility, excretion, and nutritional activities of the patient. Those scoring between 0 and 6 points are evaluated as dependent, 7–12 points semidependent, and 13–18 points as independent [8, 9]. 

The IADL Scale developed by Lawton and Brody in 1969 measures the daily activities of individuals. The IADL scale involves 8 questions regarding telephone use, food preparation, shopping, routine daily housework, laundry, transportation use, ability to use medication, and money management. On this scale, those scoring between 0 and 8 points are defined as dependent, 9–16 points as semidependent, and 17–24 points as independent [9, 10].

One of the authors (NYC) recorded all 3 scales (the KPS, the Katz ADL and the IADL) for the patients who were admitted to the palliative care outpatient unit. In the case of repeated admissions, only the scales filled during the first admission were taken into account. 

One sample Kolmogorov–Smirnov test was used for testing whether the variable follows the normal distribution in the population. Qualitative data were shown as number and percentage, and quantitative data as mean ± standard deviation. The Mann–Whitney U test was used to compare the differences of quantitative data (nonnormally distributed variables in the KPS score, Katz score, and IADL between sex groups). An independent samples t-test was used to compare the differences of quantitative data (normally distributed variables in age, length, and weight between sex groups). The Pearson correlation coefficient was used for the linear relationship between qualitative variables. In order for the IADL scale to be consistent with the other 2 scales, it was recoded to express a higher score for a good prognosis. The alpha (Cronbach) coefficient was obtained for all 3 scales.

SPSS 20.0 (Chicago, IL, USA) was used for the evaluation of all the data. The statistical significance was accepted at P < 0.05.

## 3. Results

Eighty patients were included in the study and 48 patients (60%) were male. The mean age of the patients was 61.61 ± 13.31 years, the mean height was 163.60 ± 8.44 cm and the mean weight was 63.75 ± 14.76 kg. While there was no significant difference in weight and age between the two sexes, the height difference between them was significant (P < 0.001). The most common tumors observed in the patients were stomach, lung, and colon (n: 17, 16, 10) respectively. The details of the clinical and demographic findings are shown in Table 1. 

**Table 1:** Distribution of variables by sex.

	Total	Sex		P
		Male (n = 48)	Female (n = 32)	
Age	61.61 ± 13.31	63.83 ± 10.49	58.28 ± 16.29	0.094
Height	163.6 ± 8.44	168.27 ± 6.75	156.59 ± 5.29	< 0.001
Weight	63.75 ± 14.76	64.56 ± 13.74	62.53 ± 16.32	0.550
Primary tumor sites				
Lungs	16(20)	12(25)	4(12.5)	N/A
Brain	1(1.3)	1(2.1)	0(0)	
Kidney	1(1.3)	1(2.1)	0(0)	
Colon	10(12.5)	5(10.4)	5(15.6)	
Liposarcoma	1(1.3)	1(2.1)	0(0)	
Malignant melanoma	1(1.3)	0(0)	1(3.1)	
Breast	10(12.5)	2(4.2)	8(25)	
Bladder	3(3.8)	3(6.3)	0(0)	
Mesothelioma	2(2.5)	2(4.2)	0(0)	
Stomach	17(21.3)	11(22.9)	6(18.8)	
Over	1(1.3)	0(0)	1(3.1)	
Esophagus	1(1.3)	1(2.1)	0(0)	
Pancreas	6(7.5)	2(4.2)	4(12.5)	
Prostate	2(2.5)	2(4.2)	0(0)	
Rectum	6(7.5)	4(8.3)	2(6.3)	
Cervix	1(1.3)	0(0)	1(3.1)	
Thyroid	1(1.3)	1(2.1)	0(0)	
Total	80(100)	48(100)	32(100)	

Data are given as mean, standard deviation or n (%). P < 0.05 were taken as significant.

The mean KPS score of the patients was 64.63 ± 15.34 and the mean total score of the Katz ADL was 14.66 ± 3.92. The mean total score of the IADL scale was 9.35 ± 3.92 (Table 2). There was a positive and very strong relationship between the Katz ADL total score and the KPS score (r = 0.895; P < 0.001). In addition, there was a negative correlation between the total score of the IADL scale and the KPS score (r = –0.894; P < 0.001) (Table 3). Both subdimensions of the Katz ADL and IADL scales significantly correlated with KPS scores (both P < 0.001). Cronbach’s alpha coefficient for KPS was 0.720; it was 0.912 for the 6-item Katz ADL scale and 0.947 for the 6-item IADL scale. 

**Table 2:** The sex distribution of the KPS and KATZ GYA on admission.

	Total	Sex		P
		Male (n = 48)	Female (n = 32)	
KPS a score total	64.63 ± 15.34	66.25 ± 14.82	62.19 ± 16.01	0.248
Katz GYAb score total	14.66 ± 3.92	15.25 ± 3.41	14.38 ± 3.7	0.281
TGYAc score total	9.35 ± 3.92	8.98 ± 3.65	10 ± 4.19	0.252
TGYAc score total (recoded)	14.66 ± 3.92	15.02 ± 3.65	14 ± 4.19	0.252

Data are given as mean, standard deviation or n (%). P < 0.05 were taken as significant. aKarnofsky performance scale bThe Katz index of independence in activities of daily living cThe instrumental activities of daily living scale

**Table 3:** Correlation coefficients among different scales. (Spearman’s correlation coefficient was used.)

		KPS points	Katz GYAb points	TGYAc score points (recoded)
KPSa points	r	1	0.895	–0.894
	P		<0.001	<0.001
Katz GYA points	r	0.895	1	-0.995
	P	<0.001		<0.001
TGYAc score points(re-coded)	r	–0.894	-0.995	1
	P	<0.001	<0.001	

aKarnofsky performance scale bThe Katz index of independence in activities of daily living cThe instrumental activities of daily living scale

## 4. Discussion

Numerous scales are used to evaluate palliative care patients. They have both advantages and disadvantages. It is appropriate to prefer scales that are easy to apply in practical use, easily interpretable, and available for different communities. The validity and reliability of these scales were confirmed for different societies earlier. The KPS is one of the most commonly used scales in palliative care settings. In this study, we aimed to determine the validity of the scale in Turkish palliative care patient population. In our analysis, we found a strong correlation between the KPS and the Katz ADL scale. We also found that the KPS has a negative and very strong correlation with the IADL scale. As the KPS scores of the patients decrease, the rate of performing daily life activities with or without help also decreases. As we evaluated each of the 3 scales with regard to the subdimensions, they were highly compatible. The results of our study suggest that all are reliable and applicable to patients in our country.

Many studies using various statistical analysis methods found that the KPS scoring system is a reliable measure of patient performance status. Mor et al. evaluated patients by the KPS and the Katz ADL scales and found a remarkably strong relationship between the 2 scales [4]. In a different study on cancer patients, the Pearson correlation coefficient was found to be 0.89 for the KPS and the scale was considered to be highly reliable [11]. Yates et al. found the Pearson correlation coefficient to be 0.69 when evaluating the KPS score of 52 inpatients measured independently by clinical nurses and social workers. Statistical analysis of 50 similar patients measured by a social worker in the patients’ own houses found the Pearson correlation coefficient to be 0.66. In our study, the fact that we evaluated the patients in the outpatient settings might have contributed to our high level of Pearson correlation coefficient [12].

In our study, the clinical evaluations of the patients and the administration of scales were performed by a single clinician. We think that this factor might have had an impact on the significance of the test results. A prospective study conducted on 209 patients showed that performance assessment by a clinician who used the KPS and the ECOG scales was very reliable and could be used in clinical trials [13]. In a similar study, Liem et al. asked 2 different physicians to evaluate 117 patients independently for their KPS score and observed statistically significant and perfect compliance between the scores given by both physicians. [14].

There have been many studies comparing the KPS with the Katz index of independence in activities of daily living. In our study, we found a very strong relationship between the total score of the Katz index of independence in activities of daily living and the KPS score. A similar study by Terret et al. demonstrated a weak relationship between the KPS and the physical performance test. In the physical performance test, there are entries requiring more effort, such as climbing stairs and walking 50 steps, which are different from the Katz ADL and may be the reason why the relationship was found to be weak [15].

The fact that our study was conducted in a single center and included only a limited number of patients who were admitted to our outpatient clinic may be considered a limitation. Therefore, further studies with more patients and multicentric participation are needed. 

In conclusion, performance assessment has been used for many years to assess functionality in cancer patients. It is very important that the scales to be used for this purpose are appropriate, reliable, and valid for the selected patient group. In this study, we have shown that KPS, an important performance measure, is valid in Turkish palliative cancer patients. Since the number of palliative care centers opened in Turkey has been increasing, this study might help the standardization of patients. However, further studies are needed to determine the changes in the health status over time as well as the validity and reliability of the KPS scale in different settings. 

## Acknowledgement

We thank the following individuals for their contributions to our Palliative Care Working Group: Mustafa Şahin, Nurşah Başol Kaya, Tuğba Karaca, Rıza Çıtıl, Mehmet Esen, Medine Koç, Yalçın Önder, Şahizer Eraydın, Nagehan Bayram, Nisa Nur Ayhancı, Hülya Turaç, Cem Keskin, Derya Özen, Eda Altu, Mustafa Sait Yıldız, Rabia Melek Çetin.
